# Analysis of barley mutants *ert-c.1* and *ert-d.7* reveals two loci with additive effect on plant architecture

**DOI:** 10.1007/s00425-021-03653-w

**Published:** 2021-06-20

**Authors:** Qiongxian Lu, Christoph Dockter, Nick Sirijovski, Shakhira Zakhrabekova, Udda Lundqvist, Per L. Gregersen, Mats Hansson

**Affiliations:** 1 Department of Molecular Biology and Genetics, Aarhus University, Forsøgsvej 1, 4200 Slagelse, Denmark; 2 Carlsberg Research Laboratory, J.C. Jacobsens Gade 4, 1799 Copenhagen V, Denmark; 3 Department of Biology, Lund University, Sölvegatan 35, 22362 Lund, Sweden; 4 Nordic Genetic Resource Centre (NordGen), Smedjevägen 3, 23053 Alnarp, Sweden

**Keywords:** *Erectoides*, *Hordeum vulgare*, Semidwarf, Spike density, Translocation

## Abstract

**Main conclusion:**

Both mutant *ert-c.1* and *ert-d.7* carry *T2-T3* translocations in the *Ert-c* gene. Principal coordinate analyses revealed the translocation types and translocation breakpoints. Mutant *ert-d.7* is an *Ert-c* *Ert-d* double mutant.

**Abstract:**

Mutations in the *Ert-c* and *Ert-d* loci are among the most common barley mutations affecting plant architecture. The mutants have various degrees of erect and compact spikes, often accompanied with short and stiff culms. In the current study, complementation tests, linkage mapping, principal coordinate analyses and fine mapping were conducted. We conclude that the original *ert-d.7* mutant does not only carry an *ert-d* mutation but also an *ert-c* mutation. Combined, mutations in *Ert-c* and *Ert-d* cause a pyramid-dense spike phenotype, whereas mutations in only *Ert-c* or *Ert-d* give a pyramid and dense phenotype, respectively. Associations between the *Ert-c* gene and *T2-T3* translocations were detected in both mutant *ert-c.1* and *ert-d.7*. Different genetic association patterns indicate different translocation breakpoints in these two mutants. Principal coordinate analysis based on genetic distance and screening of recombinants from all four ends of polymorphic regions was an efficient way to narrow down the region of interest in translocation-involved populations. The *Ert-c* gene was mapped to the marker interval of 2_0801to1_0224 on 3HL near the centromere. The results illuminate a complex connection between two single genes having additive effects on barley spike architecture and will facilitate the identification of the *Ert-c* and *Ert-d* genes.

**Supplementary Information:**

The online version contains supplementary material available at 10.1007/s00425-021-03653-w.

## Introduction

Plant architecture has fundamental impact on crop performance. For example, the pronounced increase in crop yield due to application of fertilizers required the simultaneous introduction of short-culm alleles in cereal elite cultivars during the “green revolution”; the *Rht-D1b* and *Rht-B1b* DELLA alleles in wheat and *sd1* alleles in rice (Hedden 2003). Also, in barley breeding, short stature became a desired trait. Loss-of-function allele of the barley dwarfing gene *Sdw1/Denso*, most likely orthologous to rice *Sd1* (Jia et al. 2009; Xu et al. 2017), has been extensively used in breeding programs in North America and Europe (Mickelson and Rasmusson 1994).

Before selection of the *sdw1/denso* “green revolution” alleles, the plant architecture of wheat and barley had already been shaped by millennia-long selection during the process of domestication and adaptation in the Neolithic revolution and human migration. Compared to wild types, both modern wheat and barley cultivars tend to have shorter rachis internodes in the spike. The mutation in the *Q* gene was suggested first to occur in tetraploid wheat during domestication (Sormacheva et al. 2015). It encodes an APETALA2 (AP2) transcription factor which regulates many domestication-related traits. Increased expression of *Q* led to higher density of spikes, and normal or reduced plant height (Faris et al. 2005; Simons et al. 2006). In barley, *HvAP2* alleles, *Zeo2* and *Zeo3*, were also found in 2-row spring UK barley as natural occurrences (Houston et al. 2013). Both alleles cause pleiotropic effects including cleistogamy (Nair et al. 2010; Houston et al. 2013), denser spike, and a haplotype-dependent change in plant height (www.nordgen.org/bgs; Franckowiak and Lundqvist 2012).

With the rise of mutagenesis techniques in cereal breeding programs, mutants induced by chemicals and radiation were collected since the early 20th century and are valuable resources for both crop improvement and biological studies. Using such collections, new mutations were, for example, found at the *Zeocriton* (*Zeo*) locus, such as *Ert-r.52*, *Ert-r.67*, *Ert-r.329*, *Ert-r.453*, *dsp.av*, *Pyr1* and *Pyr3* (Houston et al. 2013). In addition, numerous other barley genes have been described to regulate plant architecture by affecting plant stature and spike morphology (www.nordgen.org/bgs; Druka et al. 2011; Franckowiak and Lundqvist 2012). In East Asia (Japan, Korea and China), nearly 80% of 147 genotyped dwarf accessions carry the *uzu1.a* allele, derived from a selected spontaneous mutagenesis event in the brassinosteroid receptor-encoding gene *BRI1* (Zhang 1998; Jing 2000). Additionally, several independent breeding programs isolated induced dwarfing mutants representing multiple alleles in three brassinosteroid biosynthesis genes, *BRD*, *CPD* and *DIM* (Dockter et al. 2014). Using Bowman near-isogenic lines and *breviaristatum-e* (*ari-e*) original mutants, *HvDep1*, was identified as the dwarfing gene used in Scotland, affecting plant height, awn length and grain size (Wendt et al. 2016). In the Scandinavian countries, barley cultivar ’Pallas‘ and its descendants, carrying the dwarfing allele *erectoides-k.32* (*ert-k.32*), gained wide acceptance in the 1960s (Gustafsson et al. 1971; Lundqvist 2014). This allele showed effect on spike density and plant height and improved lodging resistance (Skov Kristensen et al. 2016).

The Swedish mutant collection at the Nordic Genetic Resource Centre (NordGen) maintains barley germplasm altered in two other loci, *Ert-c* and *Ert-d*, with multiple alleles affecting plant height and spike density. One allele of each, *ert-c.1* and *ert-d.7*, was introduced by recurrent backcrossing into a Bowman genetic background generating near-isogenic lines BW305 and BW306, respectively (Druka et al. 2011). Both lines were shown to have similar, rather large, introgression regions on chromosomes 2H and 3H and identical spike phenotypes (Druka et al. 2011), which contrasts with earlier results where *ert-d.7* was mapped to a 7H pericentromeric region and caused a much denser spike phenotype (Persson and Hagberg 1969). Due to these contrasting findings, we set the following objectives: (i) substantiate the identities of *ert-c.1*, *ert-d.7* and their corresponding near-isogenic lines BW305 and BW306, (ii) characterize the structure of chromosome 2H and 3H in the mutants, and (iii) explore the association between *ert-d.7* and other extreme dense *ert-d* mutants.

## Materials and methods

### Plant materials

The *ert-c.1* and *ert-d.7* mutants were both induced by X-rays in barley cultivar ‘Gull’ (Gustafsson 1947). Compared to Gull, the two mutants have semi-compact and compact spikes, respectively, caused by a reduction in rachis internode length (www.nordgen.org/bgs; Franckowiak and Lundqvist 2012). Both *ert-c.1* and *ert-d.7* are recessive mutations.

The complementation tests were conducted at Aarhus University, Flakkebjerg, Denmark. Crosses were made (Table 1) in 2014 and the F_2_ plants were phenotyped in 2015 in a semi-field area (covered outdoor area with irrigated pots). With respect to flowering time, mutants with Gull background are 2 or 3 weeks later than Bowman and Bowman near-isogenic lines. To obtain parental plants at synchronized flowering time, the *ert-c.1* and *ert-d.7* mutants as fathers were planted once a week continuously for 4 weeks. Then from the third week, Bowman near-isogenic mothers were planted continuously for 3 weeks. From each cross, F_1_ seeds from two or three spikes were grown in the greenhouse in the winter 2014–2015. Three spikes from each cross were harvested from F_1_ plants. F_2_ seeds were planted in pots in the semi-field area in summer 2015. The phenotyping was conducted at maturity after the spikes were dried, so that mis-scoring due to insufficient grain-filling was minimized. Another F_2_ population between Bowman and the original *ert-d.7* mutant was made for segregation test. F_1_ seeds were planted in the greenhouse in winter 2014–2015. A total of 160 F_2_ plants together with controls were planted and phenotyped in the semi-field area in summer 2015.

**Table 1:** Crosses for complementation tests and segregation between near-isogenic lines BW305, BW306, original mutant *ert-c.1* and original mutant *ert-d.7*

Mother	Father
BW305	BW306
BW305	*ert-c.1*
BW306	*ert-c.1*
BW305	*ert-d.7*
BW306	*ert-d.7*
*ert-d.7*	*ert-c.1*
Bowman	*ert-d.7*

Additionally, four F_2_ populations for both BW305 and BW306 were developed for genetic mapping (Table 2). BW305 populations and their respective original lines were planted and phenotyped in the greenhouse at Lund University, Sweden. BW306 populations and their respective original lines were planted and phenotyped at Lund University, and in the semi-field area at Aarhus University, Flakkebjerg, Denmark.

**Table 2:** Number of plants analyzed in F_2_ mapping populations derived from BW305 and BW306

Crosses		Phenotyping			Total
		2011 Lund	2012 Lund	2013 Flakkebjerg	
BW305	Bowman	154	126	–	280
	Barke	154	126	–	280
	Morex	154	126	–	280
	Quench	154	126	–	–
BW306	Bowman	107	–	176	283
	Barke	107	–	176	283
	Morex	99	–	176	275
	Quench	99	–	176	–
					1681

Crosses were also performed between *ert-c.1* and *ert-d.7* used as fathers and *ert-d.33*, *ert-d.43*, *ert-d.60*, *ert-d.89*, *ert-d.158*, *ert-d.307*, *ert-d.372*, *ert-d.375*, *ert-d.404* and *ert-d.420* used as mothers. The original mutants along with the F_2_ plants were grown in field conditions in Lund, Sweden, during 2020. Twenty or 40 seeds were planted of each line. Phenotyping was conducted at maturity. The spike density was measured according to Persson and Hagberg (1969) as the total length of ten rachis internodes between kernel number 5 and 15.

### Genotyping

Around 0.5 cm long leaf segments were sampled at seedling stage directly into 96-well plates. DNA was extracted with Extract-N-Amp™ (Sigma-Aldrich Co. LLC) according to manufacturer’s instructions. A ten-time dilution was used as DNA template in subsequent PCR analyses.

The introgression regions in BW305 and BW306 were previously defined primarily on 2H and 3H with a single-nucleotide polymorphism (SNP) array (Druka et al. 2011). Accordingly, SNPs in these regions are polymorphic in BW305/BW306 × Bowman populations. Primers for genotyping were designed based on the sequences of these SNPs (Close et al. 2009). SNPs used for the Bowman × *ert-d.7* population were chosen based on Bowman near-isogenic lines with Gull introgression regions (Druka et al. 2011). SNPs used in complementation tests were also obtained this way. All the markers used in this study are listed in Suppl. Table S1 and S2.

Markers 1_1530 and 1_1283 were run with the KASP system (LGC Genomics) on ABI Viia7 Real-Time PCR System (Thermo Fisher) according to manufacturer’s guide. The KASP program was modified due to low template DNA concentration: 94 °C activation 15 min, 10 cycles of 94 °C denaturation 20 s, and 61–55 °C annealing/elongation 60 s with decreasing 0.6 °C each cycle, followed by 32 cycles of 94 °C denaturation 20 s and 55 °C annealing/elongation 60 s. If the lines were not clustered well after the first run, five additional cycles of 94 °C denaturation 20 s and 57 °C annealing/elongation 60 s were added. Other markers were run with the high-resolution melting (HRM) module on the Viia 7 instrument. HRM primers were designed with Primer 3 (http://bioinfo.ut.ee/primer3-0.4.0/) with product size 60–120 bp and annealing temperature around 57 °C. If SNPs were discovered from ESTs, the EST sequences were blasted against IPK database (http://webblast.ipk-gatersleben.de/barley/) for the corresponding contig. If introns occurred, the corresponding Bowman contig was used for primer design. The reaction mix was prepared with a reduced volume of 10 µl containing 5 µl MeltDoctor HRM Master Mix (Applied Biosystems), 0.3 µl forward primer (10 µM), 0.3 µl reverse primer (10 µM), 3.4 µl water and 1 µl DNA template. The PCR program was 95 °C enzyme activation 10 min, 45 cycles of 95 °C denaturation 15 s, and 63 °C annealing/extension 45 s, followed by an HRM program: 95 °C denaturation 10 s, 60 °C annealing 1 min, high-resolution melting at a rate of 0.025 °C/s–95 °C and hold for 15 s, 60 °C annealing 15 s at the end.

### Genetic and bioinformatics analyses

The linkage map was calculated with R/qtl (Broman et al. 2003) and the map was drawn with Mapchart (Voorrips 2002). Pair-wise genetic distances were calculated with R/qtl (Broman et al. 2003), PCo was conducted in R (R Core Team 2019) and the three principal components (PCs) were visualized in 3D plots with R/rgl (Adler et al. 2016).

The primary sequences of the markers were obtained from Plant Genome and Systems Biology (PGSB) http://pgsb.helmholtz-muenchen.de/plant/barley/fpc/searchjsp/index.jsp. Physical positions of the markers were further obtained based on the blastn hit against Morex V2 reference at IPK barley blast server https://webblast.ipk-gatersleben.de/barley_ibsc/.

## Results

### Genetic identity of BW305, BW306 and the original *ert-c.1* mutant

**Fig. 1:**
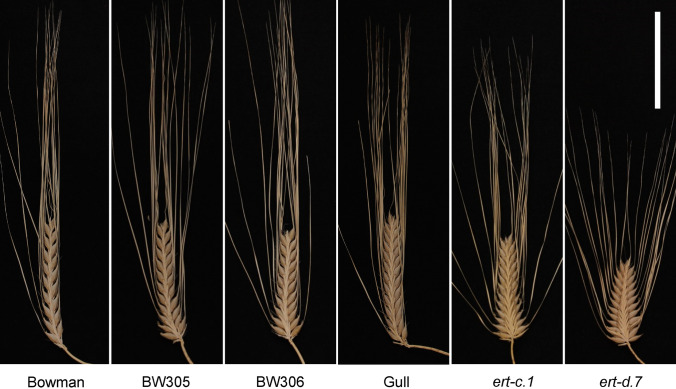
Spike phenotypes of Bowman, near-isogenic lines BW305 and BW306, Gull and original mutants *ert-c.1* and *ert-d.7*. The two *ert* mutants were induced by X-rays in the cultivar Gull in 1937 and 1941, respectively (www.nordgen.org/bgs). The near-isogenic lines BW305 and BW306, were generated from recurrent backcrosses between cultivar Bowman and *ert-c.1* and *ert-d.7*, respectively (Druka et al. 2011). Scale bar: 5 cm

The *ert-c.1* and *ert-d.7* mutants were both isolated after X-ray treatment of the barley cultivar Gull in 1937 and 1941, respectively (Gustafsson 1947). Compared to Gull, the *ert-c.1* mutant is denser at the bottom of the spike generating a pyramid-shaped phenotype, whereas the *ert-d.7* mutant is a more compact version of *ert-c.1*, i.e. pyramid-dense (Fig. 1). The compact structures are caused by reduction in rachis internode length. In contrast, the near-isogenic lines BW305 and BW306, generated from recurrent backcrosses between cultivar Bowman and *ert-c.1* and *ert-d.7*, respectively, showed similar spike phenotypes to each other (Fig. 1). According to SNP array results, BW305 and BW306 also have similar introgression regions (Druka et al. 2011). Therefore, we performed several crosses to substantiate the identities of the two near-isogenic lines and the original mutants (Table 1). Successful crosses were confirmed by testing F_1_ lines with polymorphic SNP markers (Suppl. Table S2) except for the *ert-d.7* × *ert-c.1* cross for which there is no polymorphic marker available. Here, segregating F_2_ plants were phenotyped.

**Fig. 2:**
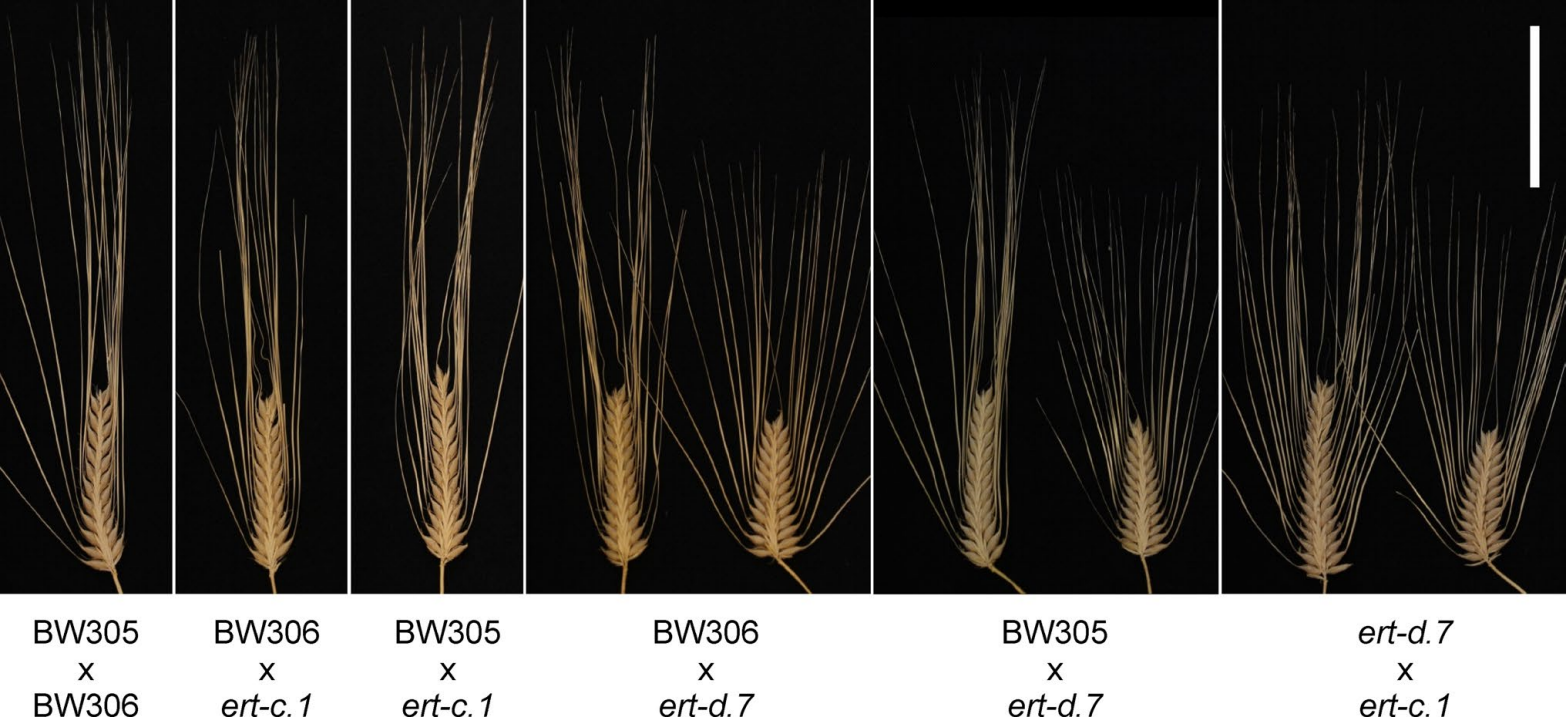
Spike phenotypes in the F_2_ generation. Two spike phenotypes with a pyramid or pyramid-dense phenotype were obtained in crosses involving *ert-d.7*, whereas only spikes with a pyramid-shaped phenotype was obtained in crosses involving *ert-c.1*. Scale bar: 5 cm

F_2_ spikes from the cross BW305 (*ert-c.1*) × *ert-c.1* were all pyramid-shaped like their parents. That is, they were compact at the bottom of the spike (Fig. 2, Table 3). This phenotype is characteristic of the *ert-c* mutants (www.nordgen.org/bgs; Franckowiak and Lundqvist 2012). A BW305 × Bowman F_2_ population segregated into 102 wild type and 32 pyramid-shaped spikes fitting a one gene inheritance with *P* value 0.76 (Table 3). We concluded that BW305 is a near-isogenic line carrying the expected *ert-c* mutation (*ert-c.1*). Furthermore, only pyramid-shaped spikes were observed in F_2_ populations of BW305 × BW306 and of BW306 × *ert-c.1* (Fig. 2, Table 3). Therefore, BW306 carries an *ert-c* mutation, which is allelic to *ert-c.1*. The segregation in a BW306 × Bowman population fitted a single-gene inheritance (Table 3). We conclude that BW305, BW306 and the original *ert-c.1* mutant are single *ert-c* mutants.

**Table 3:** The segregation of spike phenotype in F_2_ populations

Crosses	Phenotypes (no. of plants)				χ^2^	*P* value
	Wild type	Pyramid	Dense	Pyramid-dense		
BW305 × BW306	0	21	0	0	–	–
BW306 × *ert-c.1*	0	17	0	0	–	–
BW305 × *ert-c.1*	0	34	0	0	–	–
BW306 × *ert-d.7*	0	22	0	4	(3:1) 1.28	0.26
BW305 × *ert-d.7*	0	30	0	7	(3:1) 0.73	0.39
*ert-d.7* × *ert-c.1*^a^	0	4	0	4	–	–
Bowman × *ert-d.7*	83	19	35	10	(9:3:3:1) 4.74	0.19
BW305 × Bowman^b^	102	32	0	0	(3:1) 0.09	0.76
BW306 × Bowman^c^	127	38	0	0	(3:1) 0.34	0.56

^a^Not calculated because of too few plants

^b^based on 154 plants from 2011

^c^based on 176 plants from 2013

### The original *ert-d.7* line is a double mutant

**Fig. 3:**
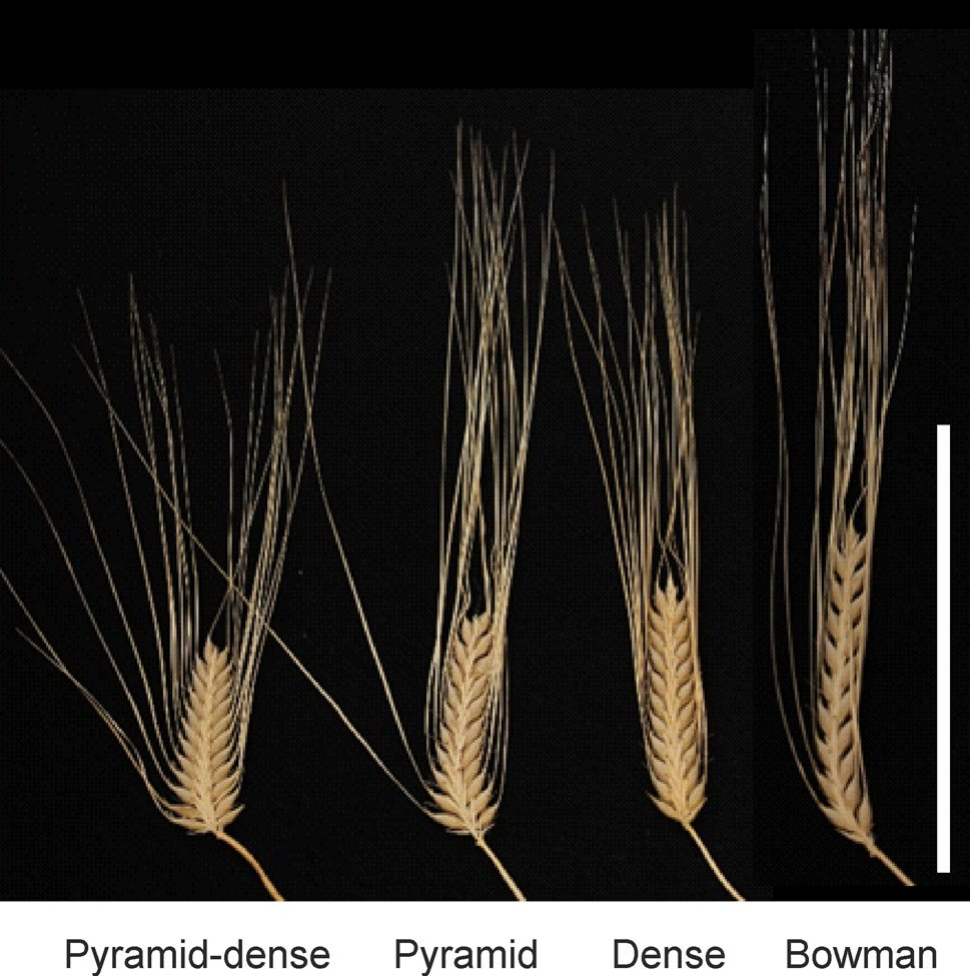
Spike phenotypes in the F_2_ generation from a cross between Bowman and *ert-d.7*. Spikes with a pyramid-dense, pyramid or dense phenotype were obtained in addition to spikes with a Bowman phenotype. Scale bar: 10 cm

Three crosses were made with the original *ert-d.7* mutant line for complementation tests; two as father crossed with BW305 and BW306, and one as mother crossed with *ert-c.1* (Table 1). In each F_2_ population, there were two different types of compact spikes. One resembled the *ert-c.1* mutant phenotype, being only compact at the bottom of the spike, i.e. pyramid-shaped (Fig. 2). The other type was pyramid-shaped but also dense along the entire spike, which resembled the phenotype of the original *ert-d.7* mutant, i.e. pyramid-dense (Fig. 1). The results suggested segregation of an *ert-d.7* mutation in an *ert-c* genetic background. Accordingly, the original *ert-d.7* mutant line appears to be a double mutant carrying an *ert-c* mutation in addition to the *ert-d.7* mutation. This was confirmed in a Bowman × *ert-d.7* F_2_ population in which wild type spikes and three different compact spikes were observed, denoted pyramid-dense, pyramid and dense (Fig. 3, Table 3). The dense spike is suggested to represent the *ert-d.7* phenotype in a Bowman genetic background, while the pyramid spike is the *ert-c* phenotype and the pyramid-dense is the *ert-c ert-d.7* double mutant phenotype. The segregation ratio gave a Pearson’s chi-square χ^2^ = 4.74 (*P* value = 0.19). Thus, the hypothesis was accepted that the two genes are independent (Table 3). The observation of a double mutant phenotype in the original *ert-d.7* mutant and in the segregating Bowman × *ert-d.7* F_2_ population demonstrated an additive effect of the two genes on plant architecture.

### Mapping of *Ert-c* in relation to *T2-T3* translocations

Genetic mapping of *Ert-c* was initially conducted in F_2_ populations of BW305 × Bowman with 154 plants, BW306 × Bowman with 176 plants, and Bowman × *ert-d.7* with 160 plants. The linkage map indicated that both of the two *ert-c* alleles are associated with markers on both chromosome 2H and 3H, which suggested translocation events. To investigate this, principal coordinate analyses were conducted as suggested by Farre et al. (2011). However, pair-wise genetic distance was used instead of similarity. In the next step, the candidate region for the *Ert-c* gene was refined by screening all segregating lines with flanking markers. Finally, the recombinants were further screened with all associated markers on 2H and 3H.

**Fig. 4:**
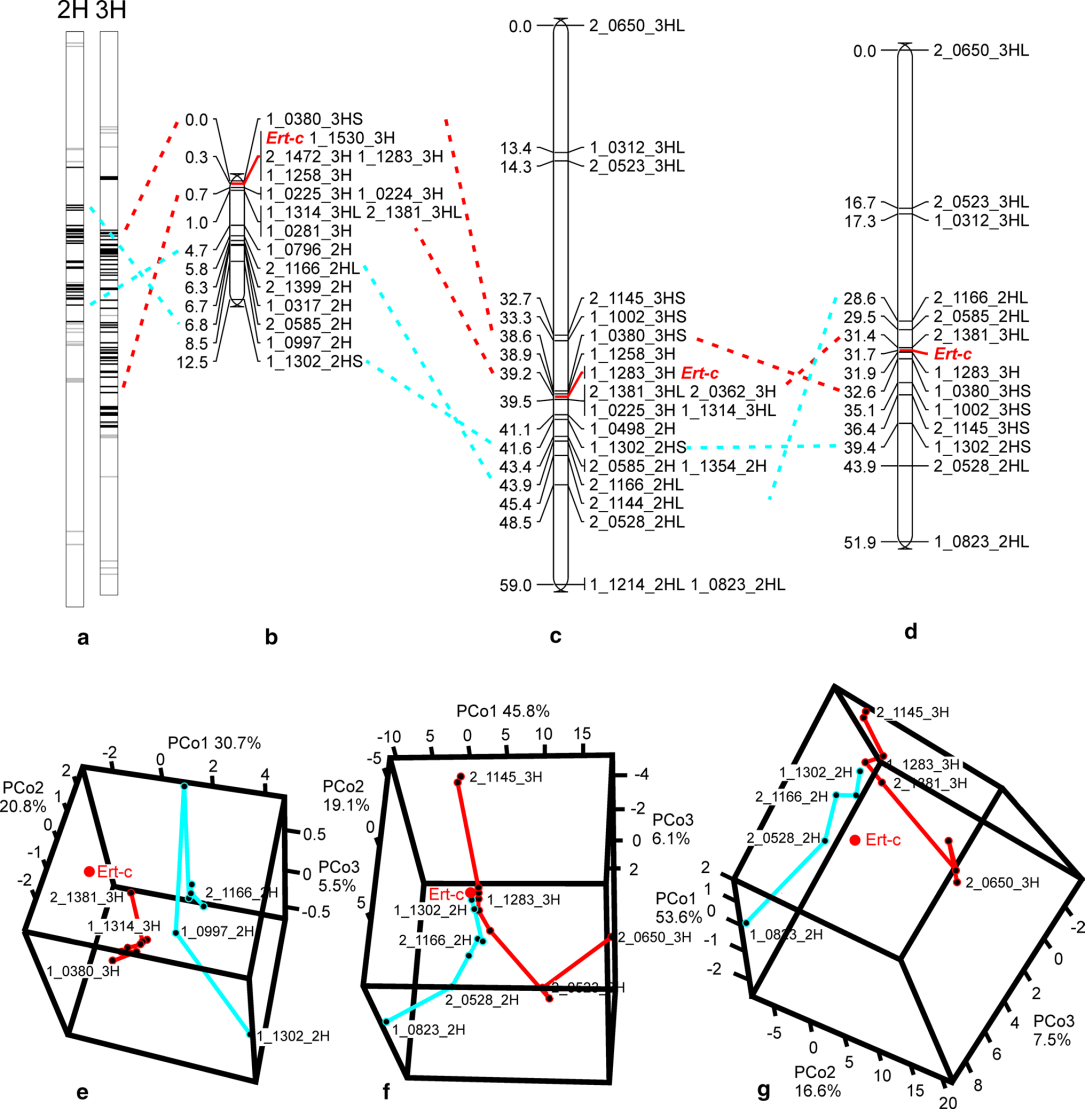
Genetic maps of the *Ert-c* locus. **a** The introgression regions detected by Druka et al. (2011) between BW305/BW306 and Bowman. The black bars indicate the location of markers defining the introgression regions in both BW305 and BW306. The gray bars indicate markers only found in BW306. **b-g** The linkage map and the 3D plot of pair-wise genetic distance among all the linked markers (principal coordinate analysis) in a BW305 × Bowman F_2_ population **(b, e)**, in a BW306 × Bowman F_2_ population (**c, f**) and in a Bowman × *ert-d.7* F_2_ population (**d, g**). In the 3D plots, the markers are connected with lines according to the chromosome order in the consensus map (Druka et al. 2011) and the proportion of variance captured is given as a percentage for each PCo dimension

In linkage mapping, *Ert-c* was mapped between the markers 1_0380–1_0225 on chromosome 3H near the centromere using both F_2_ populations of BW305 × Bowman and of BW306 × Bowman (Fig. 4b, c). In the Bowman × *ert-d.7* F_2_ population, *Ert-c* was co-segregating with chromosome 3H markers 1_1283 and 2_1381 and also the 2H markers 1_1302 and 2_0585 (Fig. 4d). Although *Ert-c* did not co-segregate with 2H markers in mapping populations of BW305 and BW306, it was still closely linked to them. This indicated that *T2-T3* translocations are involved in BW305, BW306 and the *ert-d.7* mutant. In addition, by adding few markers at a time to the chromosome, the map order varied constantly especially in BW306 × Bowman and Bowman × *ert-d.7* populations where polymorphic markers span bigger regions and were distributed unevenly along the two chromosomes. This also indicated problematic mapping order. In conventional genetic mapping, pair-wise recombination frequencies of all markers are first estimated, then linkage groups are formed and markers from the same linkage group are placed into linear order. This will naturally cause a problem when a translocation is involved since markers from both chromosomes will be placed in one linkage group. Therefore, we converted pair-wise recombination frequencies into pair-wise genetic distances illustrated in 3D plots (Fig. 4e–g). By doing so, we not only showed associations between markers around the translocation point but also avoided placing all 2H and 3H markers into one linear linkage group. Despite a different marker order in the conventional linkage maps, the 3D maps showed similar patterns in the BW306 × Bowman (Fig. 4f) and Bowman × *ert-d.7* (Fig. 4g) populations. The 3H marker 1_1283 was close to 2H marker 1_1302 based on pair-wise genetic distance, while the rest of 2H markers and 3H markers towards the telomere were gradually diverging. The closest points between 3 and 2H in the BW305 × Bowman population were 1_1314 and 1_0997 (Fig. 4e).

To refine the candidate region, we performed an initial screening of available F_2_ plants from segregating populations (Table 2) with markers flanking the *Ert-c* locus to identify a small set of recombinant plants, which were further screened with all associated markers on 2H and 3H (Suppl. Table S1). The markers 1_0225 and 1_0380 were identified above as closely linked to *Ert-c* (Fig. 4b, c). However, 1_0225 was only polymorphic in BW305/BW306 × Bowman populations, while most of the other markers in the 3H interval were polymorphic in BW305/BW306 × Bowman and BW305/BW306 × Barke populations (Suppl. Table S1). Therefore, the closely linked 1_1314 marker was used instead of 1_0225 together with 1_0380 for the initial screening. In addition, since there were co-segregating markers on 2H in the Bowman × *ert-d.7* population (Fig. 4d), 2H markers 1_1302 and 2_1166 were used as well in search for recombinant lines. In the Bowman × *ert-d.7* population, all markers in the candidate region, and some markers on 2H co-segregated with *Ert-c* which did not provide more information. Therefore, this population was excluded from fine mapping. The Quench populations were also excluded from further screening due to low polymorphism in the region (Suppl. Table S1). The majority of markers in the 2H region are polymorphic in Morex populations rather than in the Barke populations and most 3H markers are polymorphic in Barke populations rather than Morex populations (Suppl. Table S1). So the recombinants from 1_0380 and 1_1314 screening in Barke populations were only used for 3H marker analyses, while the recombinants from 1_1302 and 2_1166 screening in Morex populations were only used for 2H marker analyses. The recombinants from Bowman populations were used for the markers from both chromosomes. In total, 1262 out of 1681 F_2_ plants from segregating populations were analyzed (Tables 2 and 4). The remaining plants had either missing or unreliable phenotypes and hence were excluded from further analyses. According to 3D plots in the principal coordinate analyses (Fig. 4e, f), the *ert-c* mutations from BW305 and BW306 are most likely from different mutation events. To avoid any unexpected consequences from different chromosome rearrangement, fine mapping was conducted separately in BW305 and BW306 populations. In BW305 populations, the candidate region was narrowed down to the interval of 2_0801 to 1_0224 on 3H and eight markers were co-segregating with *Ert-c* (Table 4). All 2H markers showed recombinations. Thus, the 2H markers were not closer to *Ert-c* compared to the co-segregating 3H markers. In BW306 populations, the candidate region was in the interval 1_0380 and 1_0225. Also in the BW306 population, all 2H markers showed recombinations. We conclude that the interval between 2_0801 and 1_0224 is the narrowest for *Ert-c*. Marker 2_0801 is located at bp 348,705,578 and marker 1_0224 at bp 390,615,716 according to the Morex V2 assembly (Monat et al. 2019). Thus, *Ert-c* is located in a 42 Mbp interval on chromosome 3H containing 376 genes (160 high-confidence genes).

**Table 4:** Recombinant screening with markers in *Ert-c* candidate region and their physical position^a^

Marker^b^	Chr	BW306 ×				BW305 ×				Physical position
		Bowman	Barke	Bowman	Morex	Bowman	Barke	Bowman	Morex	
		1_0380^c^1/566^d^	1_1314 3/566	1_1302 2/283	2_1166 12/558	1_0380 3/474	1_1314 2/474	1_1302 19/280	2_1166 18/560	
1_1302	2H	0	0	2	1(3)	1(2)	2	19	6(9)	chr2H:80,624,539
1_0997	2H	0	0	1	3(3)	0(2)	2	6	6(9)	chr2H:112,663,998
1_0796	2H	0	1(1)^e^	1	10	0(2)	2	7	9	chr2H:164,050,763
1_0602	2H	0	1(1)	1	10	0(2)	2	6	9	chr2H:168,991,960
2_1286	2H	0	1(1)	1	10	0(2)	2	6	9	chr2H:180,576,801
2_0476	2H	0	1(1)	1	3(3)	0(2)	2	6	6(9)	chr2H:115,671,669
1_0070	2H	0	1(1)	1	10	0(2)	2	6	9	chr2H:173,480,594
1_0012	2H	0	1(1)	1	10	0(2)	2	6	9	chr2H:189,270,150
2_0039	2H	0	1(1)	1	10	0(2)	2	6	9	chr2H:203,689,667
2_0669	2H	0	1(1)	1	10	0(2)	2	6	9	chr2H:364,454,153
2_0458	2H	0	1(1)	1	10	0(2)	2	6	9	chr2H:208,375,495
2_0417	2H	0	1(1)	1	10	0(2)	2	6	9	chr2H:221,627,205
2_0032	2H	0	1(1)	1	10	0(2)	2	6	9	chr2H:365,198,431
1_1354	2H	0	1(1)	1	10	0(2)	2	6	9	chr2H:215,895,944
1_0317	2H	0	1(1)	1	10	0(2)	2	6	9	chr2H:342,925,311
2_0690	2H	0	1(1)	1	3(3)	–	–	–	–	chr2H:525,128,564
2_1399	2H	0	1(1)	1	10	0(2)	2	6	10	chr2H:478,645,444
2_0585	2H	0	1(1)	1	10	0(2)	2	6	9	chr2H:442,060,504
2_1166	2H	0	1(1)	1	12	0(2)	2	6	18	chr2H:517,863,521
1_0380	3H	1	0	0	0(3)	3	0	1	0(9)	chr3H:125,195,845
1_1258	3H	0	0	0	0(3)	1	0	0	0(9)	chr3H:179,020,686
2_0002	3H	0	0	0	0(3)	1	0	0	0(9)	chr3H:376,617,629
2_0801	3H	0	0	0	0(3)	1	0	0	0(9)	chr3H:348,705,578
2_1129	3H	0	0	0	0(3)	0	0	0	0(9)	chr3H:181,511,027
2_0288	3H	0	0	0	0(3)	0	0	0	0(9)	chr3H:337,603,488
1_0966	3H	0	0	0	0(3)	0	0	0	0(9)	chr3H:383,547,550
2_1472	3H	0	0	0	0(3)	0	0	0	0(9)	chr3H:339,775,259
2_1435	3H	0	0	0	0(3)	0	0	0	0(9)	chr3H:369,794,368
2_0486	3H	0	0	0	0(3)	0	0	0	0(9)	chr3H:384,118,435
1_1530	3H	0	0	0	0(3)	0	0	0	0(9)	chr3H:350,149,112
1_1283	3H	0	0	0	0(3)	0	0	0	0(9)	chr3H:337,029,655
1_0224	3H	0	0	0	0(3)	0	1	1	0(9)	chr3H:390,615,716
1_0225	3H	0	1(1)	0	1(3)	0(2)	1	1	0(9)	chr3H:411,660,833
1_0653	3H	0	1(1)	0	6	0(2)	1	1	4	chr3H:439,953,129
1_0281	3H	0	1(1)	0	6	0(2)	2	2	4	chr3H:455,306,801
1_1314	3H	0	3	0	6	0	2	2	4	chr3H:485,299,247

^a^Physical position based on the Morex V2 assembly (Monat et al. 2019)

^b^Markers are listed based on genetic distance (Druka et al. 2011)

^c^Flanking marker used in initial screen to find recombinant lines

^d^Number of recombinants out of total number of screened plants

^e^The figures in brackets mean number of recombinant plants that were screened with corresponding marker (row) out of total recombinants from the flanking marker (column). The remaining recombinants from the flanking marker were not screened due to no polymorphism for the corresponding marker

### Double mutations in other *ert-d* mutant lines

**Fig. 5:**
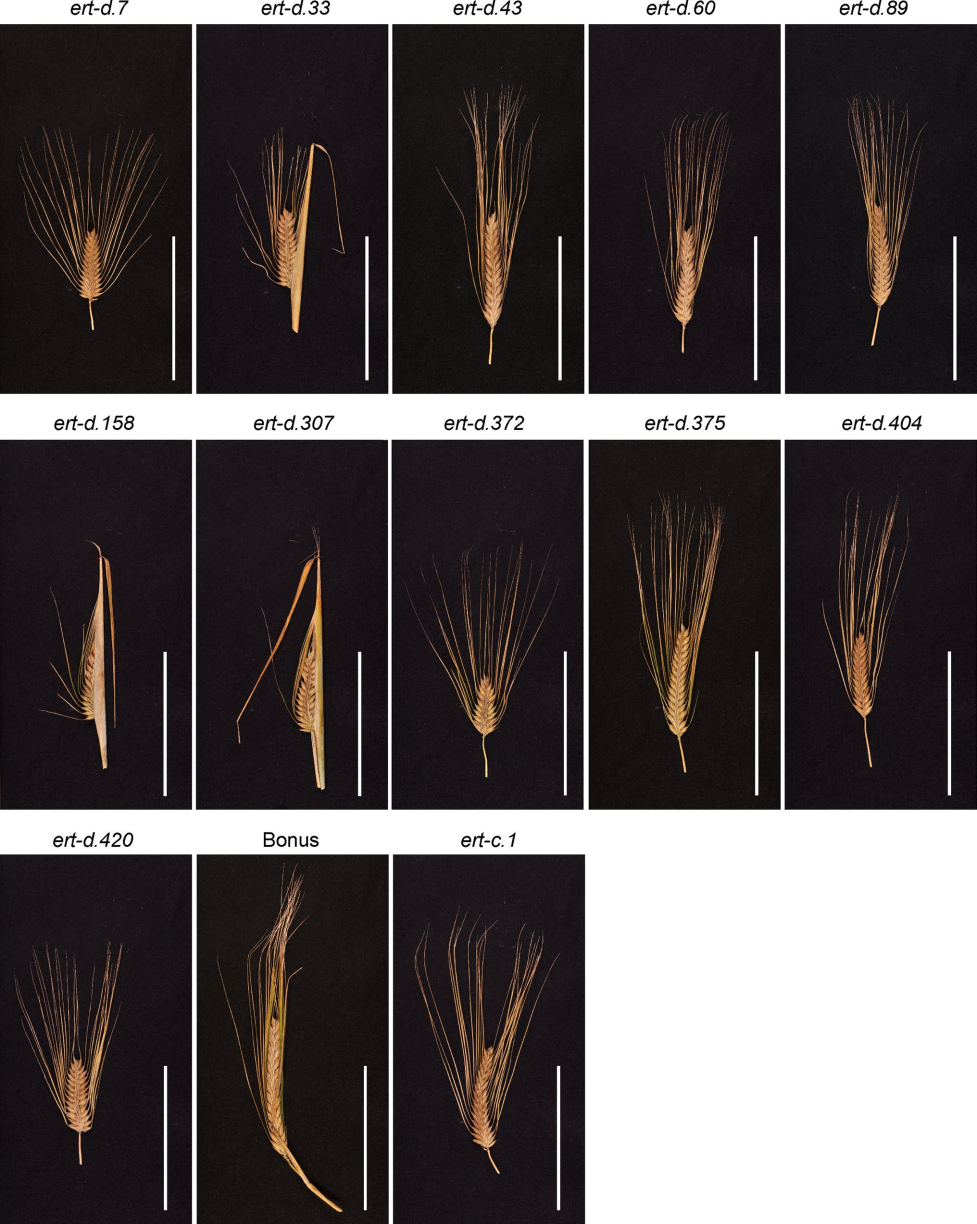
Spike phenotypes of *ert-d* mutants. Scale bars: 10 cm

There is a considerable phenotypic variation among the available 27 *ert-d* mutants. The large phenotypic variation was also noted by Persson and Hagberg (1969), who identified two distinct groups; one group consisting of extremely compact mutants and one consisting of compact mutants. Combining plant height and spike density, the *ert-d* mutants also cluster in two groups (Suppl. Fig. S1). To evaluate if this variation is due to the presence of an additional mutation in *Ert-c* like in the case of mutant *ert-d.7*, a selection of ten *ert-d* mutants were further analyzed (Fig. 5). The mutants were selected from both groups identified by Persson and Hagberg. The extremely compact group consisted of mutants *ert-d.33*, *ert-d.158*, *ert-d.307*, *ert-d.372* and *ert-d.420* (Table 5). Their spike length, spike density and culm length were in the range of 49.6–58.4 mm, 15.6–17.6 mm and 53.9–55.5 cm, respectively. Concerning spike length and culm length, these measures are approximately 60% compared to the cultivar Bonus, while spike density is 51%. The compact group consisted of mutants *ert-d.43*, *ert-d.60*, *ert-d.89*, *ert-d.375* and *ert-d.404* (Table 5). Their spike length, spike density and culm length were in the range of 65.1–76.5 mm, 22.9–26.7 mm and 64.6–65.7 cm, respectively. Concerning spike length and spike density, these measures are approximately 75% compared to the cultivar Bonus, while culm length is 91% (Table 5). Mutant *ert-d.7* appeared to be intermediate concerning spike length (62.1 mm) and spike density (19.8 mm) but was taller than Bonus (culm length 82.4 cm) (Table 5). Bonus, *ert-d.7*, *ert-d.33*, *ert-d.43*, *ert-d.60*, *ert-d.89*, *ert-d.158* and *ert-d.307* were also studied by Persson and Hagberg (1969). There is a good match between our measures and theirs except for *ert-d.158*, which belonged to the compact group in their study and to the extremely compact group in our study. The difference indicates that we studied a different accession of *ert-d.158*.

**Table 5:** Phenotypic characterization of *ert-d* mutants

Name	Spike length (mm)^c^	Spike density (mm)^d^	Culm length (cm)
*ert-d.7*	62.1 ± 2.1	19.8 ± 0.30	82.4 ± 0.72
*ert-d.33*^a^	58.4 ± 1.4	17.6 ± 0.22	55.5 ± 0.93
*ert-d.43*^b^	71.9 ± 5.4	26.7 ± 0.32	64.6 ± 0.85
*ert-d.60*^b^	76.5 ± 3.3	24.6 ± 0.36	65.4 ± 0.91
*ert-d.89*^b^	67.0 ± 3.8	23.6 ± 0.29	65.0 ± 0.86
*ert-d.158*^a^	54.0 ± 1.7	15.6 ± 0.37	53.9 ± 1.3
*ert-d.307*^a^	56.2 ± 2.6	16.0 ± 0.22	54.4 ± 1.1
*ert-d.372*^a^	49.6 ± 2.7	17.0 ± 0.31	54.8 ± 1.2
*ert-d.375*^b^	72.8 ± 3.5	22.9 ± 0.27	65.7 ± 0.86
*ert-d.404*^b^	65.1 ± 3.1	23.0 ± 0.48	65.1 ± 0.84
*ert-d.420*^a^	52.1 ± 2.0	16.8 ± 0.29	54.7 ± 1.4
*ert-c.1*	77.3 ± 1.8	22.2 ± 0.31	75.0 ± 0.60
Bonus	90.7 ± 1.8	32.5 ± 0.27	71.6 ± 0.50

^a^Mutants belonging to the extremely compact group

^b^Mutants belonging to the compact group

^c^Averages ± standard deviation

^d^The quantitative measure of spike density was the length of ten rachis internodes between the base of kernel number 5 and the base of kernel number 15

**Fig. 6:**
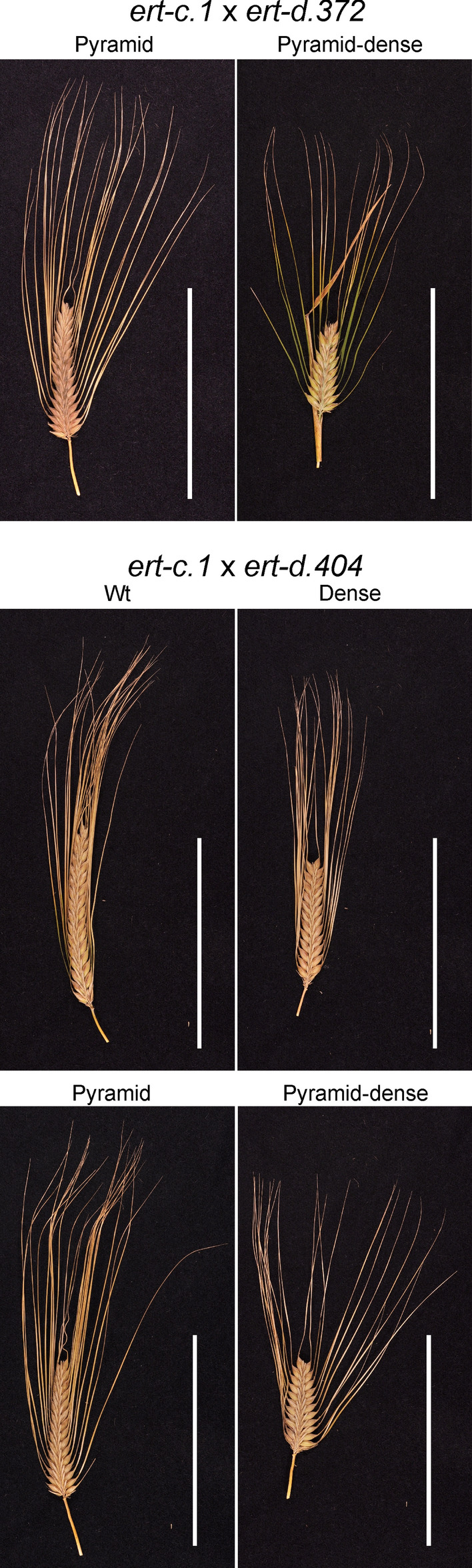
Spike phenotypes in the segregating F_2_ generation from a cross between *ert-c.1* and *ert-d.372*, and *ert-c.1* and *ert-d.404*. The presence of wild type spikes in the *ert-c.1* × *ert-d.404* population demonstrates that *ert-d.404* does not carry an *ert-c* mutation. This is in contrast to the *ert-c.1* × *ert-d.372* population where no wild type spikes were seen, which strongly suggests that *ert-d.372* is a double *ert-c ert-d.372* mutant. Scale bars: 10 cm

To investigate the possibility of *ert-c ert-d* double mutations in the ten *ert-d* mutant lines, crosses were performed between *ert-c.1* (father in the crosses) and *ert-d.33*, *ert-d.43*, *ert-d.60*, *ert-d.89*, *ert-d.158*, *ert-d.307*, *ert-d.372*, *ert-d.375*, *ert-d.404* and *ert-d.420* (mothers). Mutants carrying an additional mutation in *Ert-c* were expected to display a segregation pattern similar to *ert-d.7* in the F_2_ generation when crossed to *ert-c.1*, i.e. pyramid and pyramid-dense spikes in a 3:1 ratio. In contrast, *ert-d* mutants not carrying an additional *ert-c* mutation, were expected to show spikes with a wild type phenotype mixed with dense, pyramid and pyramid-dense spikes in a 9:3:3:1 ratio. Among the ten *ert-d* lines, only *ert-d.372* is an *ert-c ert-d* double mutant. All other crosses generated spikes with a wild type phenotype in the F_2_ generation demonstrating that they are not carrying an additional *ert-c* mutation (Fig. 6, Table 6).

**Table 6:** Segregation of spike phenotype in F_2_ populations from crosses between *ert-d* mutants and *ert-c.1*

Crosses	Phenotypes (no. of plants)				χ^2^	*P* value
	Wild type	Pyramid	Dense	Pyramid-dense		
*ert-c.1* × *ert-d.7*	0	11	0	4	(3:1) 0.022	0.88
*ert-c.1* × *ert-d.33*	6	5	5	0	(9:3:3:1) 4.67	0.20
*ert-c.1* × *ert-d.43*	18	11	6	0	(9:3:3:1) 5.38	0.15
*ert-c.1* × *ert-d.60*	24	6	6	1	(9:3:3:1) 1.49	0.68
*ert-c.1* × *ert-d.89*	17	9	9	2	(9:3:3:1) 1.97	0.58
*ert-c.1* × *ert-c.158*	7	6	6	0	(9:3:3:1) 5.80	0.12
*ert-c.1* × *ert-d.307*	15	3	2	0	(9:3:3:1) 3.47	0.32
*ert-c.1* × *ert-d.372*	0	15	0	5	(3:1) 0.00	1.00
*ert-c.1* × *ert-d.375*	18	6	6	2	(9:3:3:1) 0.00	1.00
*ert-c.1* × *ert-d.404*	14	9	8	1	(9:3:3:1) 3.56	0.31
*ert-c.1* × *ert-d.420*	8	5	4	1	(9:3:3:1) 1.36	0.71

## Discussion

### Phenotypical assessment and the identities of the lines

The *ert-c.1* and *ert-d.7* mutants were isolated from the barley cultivar Gull after X-rays treatment (www.nordgen.org/bgs). Their spikes have a compact appearance caused by a reduction in rachis internode length. They have historically been mapped to chromosomes 3HL and 7HS, respectively (www.nordgen.org/bgs), but a later study using near-isogenic lines BW305 (*ert-c.1*) and BW306 (made from the original *ert-d.7* mutant) suggested that both mutations are located on chromosomes 3H or 2H (Druka et al. 2011), which can be interpreted as they would be allelic. In the present study we analyzed different populations derived from crosses of the *ert-c* and *ert-d* mutants to understand their nature. Segregation analysis of the original *ert-d.7* mutant in an *ert-c.1* genetic background demonstrated that *ert-d.7* is an *ert-c ert-d* double mutant. Only the *ert-c* mutation remains in BW306. Moreover, a *T2-T3* translocation associated with *Ert-c* was detected in the *ert-d.7* mutant, as well as in the *ert-c.1* mutant. The translocation in the *ert-c.1* mutant was initially detected by karyotype analysis (Hagberg and Tjio 1951). After six generations of backcrosses, the introgression regions of BW305 and BW306 are still split on chromosomes 2H and 3H (Druka et al. 2011), which further indicate *T2-T3* translocations in the *ert-c* alleles of BW305 and BW306. Using polymorphic markers in the region, we confirmed the translocations by linkage mapping and principal coordinate analyses through the fact that markers from both 2H and 3H are associated with *Ert-c*. According to the principal coordinate analyses, BW306 and the original *ert-d.7* mutant have similar diverging pattern, which strongly support an identical reciprocal translocation that is different from that in BW305 and *ert-c.1*. In addition, the data also indicates different translocation breakpoints. In BW306 × Bowman and Bowman × *ert-d.7* populations, 1_1283 and 1_1302 are the closest SNP markers joining chromosomes 3H and 2H. In the BW305 × Bowman population, the joining SNP markers are 1_1314 and 1_0997. The difference between BW305 and the *ert-d.7* mutant/BW306 indicates that the *ert-c* mutation in BW305 and *ert-c.1* is a different mutation event from that in BW306 and the *ert-d.7* mutant. Analysis of ten additional *ert-d* mutants, demonstrated that only *ert-d.372* is an *ert-c ert-d* double mutant like *ert-d.7*. Future analyses will show whether *ert-d.372* is carrying a *T2-T3* translocation.

### Mapping in translocation-involved populations

Conventional linkage mapping generates only one-dimensional maps, which cause pseudo-linkage when translocations are involved. Livingstone et al. (2000) reported that the variance in the genetic distance between any two markers on the same segment was on average tenfold higher than that of markers on different segments and they were able to separate the markers distal to the translocation breakpoints by comparing these variances. Durrant et al. (2006) introduced QuadMap with modifications of this method. However, both methods are based on simulated data with all the markers spanning whole chromosomes at a relatively larger and more even distance. The polymorphic regions in the current study involve relatively small parts of the chromosome which would probably influence the variance estimation between markers from different chromosomes, especially in the BW305 × Bowman population. Farre et al. (2011) suggested to perform principal coordinate analyses and then divide the studied double-haploid population into normal type and translocated type according to the origin of the alleles at the translocation breakpoints. In the current study, this is not an option. In our F_2_ populations, there are also heterozygous plants with one translocation gamete from alternative segregation and one normal gamete. These heterozygous plants, which constitute about half of the F_2_ population would have to be excluded if applying the method of Farre et al. (2011). Therefore, we first applied principal coordinate analyses according to Farre et al. (2011) to investigate the possible translocation types and translocation breakpoints. Then, we screened 3H recombinants with 2H markers to see if they were closer and vice versa. This approach is independent from the genetic map and the translocation type. In the principal coordinate analyses, however, genetic distance was used instead of similarity which also includes the information of heterozygous genotypes. In principal coordinate 3D plots, the location of the *Ert-c* gene is not close to either of the chromosomes due to its dominant nature as a marker. Compared to co-dominant markers, dominant markers cannot distinguish heterozygous dominance from homozygous dominance, which largely influenced the estimation of genetic distance.

### Genetic mapping and candidate gene selection

Many barley genes have been identified through fine mapping (Hicks et al. 2001; Chono et al. 2003; Akagi et al. 2004; Zakhrabekova et al. 2012; Schneider et al. 2016; Matyszczak et al. 2020). In the last decade with fast development of sequencing technologies, many plant genomes have been sequenced including barley (Mascher et al. 2017). This resource facilitates positional cloning since genetic mapping data can be directly translated into the physical map revealing all genes located between flanking markers (Hansson et al. 2018). The barley spike density gene *Zeo* was cloned in this way. The authors first mapped the locus in a region containing 29 barley gene models, where one possible candidate gene immediately drew attention due to its function in cereal spike development (Houston et al. 2013). Another successful example is the cloning of the plant architecture gene *Ert-m* (Zakhrabekova et al. 2015). In that study, one candidate gene in the mapped interval was orthologous to *ERECTA* in Arabidopsis which had already been cloned. The cloning of early-maturity gene *Mat-a* was performed in a similar way (Zakhrabekova et al. 2012). All these successful examples have mostly relied on the synteny to genes of known functions in other species.

In the current study, the *Ert-c* gene was mapped to a segment on the long arm of chromosome 3H near the centromere. Here, recombination frequencies are low in centromeric as well as in translocation regions, which make the cloning of *Ert-c* difficult by marker assisted methods. Although 1262 plants were screened, many markers still remained co-segregating with *Ert-c* in the physical position ranging from 349 to 391 Mbp on chromosome 3H. We took a closer look for candidate genes at this 42 Mbp interval comprising 376 genes of which 160 are high-confidence genes. Through BLAST searches in NCBI, there is no apparent indication that any of them are involved in development of spike architecture. We are currently trying to identify *Ert-c* by whole genome sequencing, which is independent of gene order and location relative to regions with reduced recombination.

## Conclusion

In the present study, we found an association between the *Ert-c* gene and *T2-T3* translocations, i.e. translocations between chromosomes 2H and 3H, in both *ert-c.1* and *ert-d.*7 mutants. Our results demonstrate that the original *ert-d.7* mutant carries mutations in two loci, both *ert-d* and *ert-c*. The *ert-c* locus was mapped into an interval of 42 Mbp comprising 376 genes, which will facilitate the further analysis towards identification of the mutated gene. The additive effect of the two genes on spike architecture suggests that they are involved in two different molecular pathways or are active at different timepoints during the development of the emerging spike. The *Ert-c* gene regulates the distance between the rachis nodes at the bottom of the spike, whereas *Ert-d* regulates the distances along the entire spike. This might be explored in future plant breeding where an extensive knowledge of genes and gene functions, combined with large sets of genetic markers, will allow detailed design of spike architecture.

### *Author contribution statement*

QL, NS, SZ and MH performed crosses. QL performed genotyping. QL and MH prepared figures. QL, CD, NS, SZ, UL, PLG and MH performed phenotyping, wrote the article, and contributed to the discussion.

## Supplementary Information

Below is the link to the electronic supplementary material.

Supplementary file1 (DOCX 96 kb)
