# Supplementary material for: Analysis of barley mutants ert-c.1 and ert-d.7 reveals two loci with additive effect on plant architecture
Source: Planta. 2021 Jun 20;254(1):9. doi: 10.1007/s00425-021-03653-w (PMC8215040; doi:10.1007/s00425-021-03653-w)

**Suppl. Table S1** Markers used for mapping of *Ert-c*.

| SNP | Chr. | Genetic position  (cM)^1^ | Physical position  (bp)^2^ | Primer sequences 5' to 3' | | Size (bp) | BW306 | | | | BW305 | | | |
| --- | --- | --- | --- | --- | --- | --- | --- | --- | --- | --- | --- | --- | --- | --- |
|  |  |  |  | Forward | Reverse |  | Bowman | Barke | Morex | Quench | Bowman | Barke | Morex | Quench |
| 1_0498 | 2H | 81.43 | 68633857 | AAACGGCCACAACCTTACTG | TAATGTGGGTGGAGGCTTTG | 78 | X^3^ | ND^4^ | ND | ND | -^5^ | ND | ND | ND |
| 1_1302 | 2H | 86.79 | 80624539 | GTAGCTAGTCTTGCCAGGGA | TTTCTTGCAGGGTTAATTTCCG | 95 | X | - | - | - | X | - | - | - |
| 1_0997 | 2H | 94.48 | 112663998 | GACACAAAAGGCACCCAGTT | TCATCCTGTGATCCTGTCCA | 98 | X | - | - | - | X | - | - | - |
| 1_0796 | 2H | 95.53 | 164050763 | ACGAAATAGATGGATGGGTGC | GCTCCGACTCTCCCTGATG | 83 | X | - | X | - | X | - | X | - |
| 1_0602 | 2H | 95.53 | 168991960 | GGGTTCCCAAGGTAATCGAGA | GAGTTCAAGGAGCCCGTCT | 77 | X | - | X | - | X | - | X | - |
| 2_1286 | 2H | 96.47 | 180576801 | TTCCTTCACCATTGCTCCCT | AGGAGGCGAGAACTTTGGAG | 93 | X | - | X | - | X | - | X | - |
| 2_0476 | 2H | 96.47 | 115671669 | CATTGGAGAGCACGCAGATC | AGGGGAACTGATCACTTCCG | 108 | X | - | - | - | X | - | - | - |
| 1_0070 | 2H | 96.47 | 173480594 | CTCATCTGGATCGAGGTGCT | CAGCGGGTCGAAGTAGGAG | 108 | X | - | X | - | X | - | X | - |
| 1_0012 | 2H | 96.47 | 189270150 | CAGGTTTGAGCGTCCAGGAG | TGGATAGAACATTCCCGCTACT | 74 | X | - | X | - | X | - | X | - |
| 2_0039 | 2H | 97.41 | 203689667 | GATCCCATGTTCACGTCTCATC | AGGTGATGGATGCTGGTAGTAC | 81 | X | - | X | - | X | - | X | - |
| 2_0669 | 2H | 98.35 | 364454153 | GGTTTGATCCCATGTTCACGT | AGGTGATGGATGCTGGTAGT | 86 | X | - | X | - | X | - | X | - |
| 2_0458 | 2H | 98.35 | 208375495 | TTGAAGGCCGCATTTCACTC | TCCTCTTGCTACCAGGTTCG | 58 | X | - | X | - | X | - | X | - |
| 2_0417 | 2H | 98.35 | 221627205 | TCATCACAGAGCATTAGGAACA | CATCTGAATGTCGGCCTGTA | 92 | X | - | X | - | X | - | X | - |
| 2_0032 | 2H | 98.35 | 365198431 | ATCAACCCCACCACCAAGAA | GTAGGACTGCTGGCCGAG | 93 | X | - | X | - | X | - | X | - |
| 1_1354 | 2H | 98.35 | 215895944 | GCTGCACCAACACAAAACAC | GCACTGAAGCTTCTCGGTAA | 69 | X | - | X | - | X | - | X | - |
| 1_0317 | 2H | 98.35 | 342925311 | CGTTGGGTCATCTCTGCAAA | CAAGTTAATGACGGAGCCTGT | 83 | X | - | X | - | X | - | X | - |
| 2_0690 | 2H | 102.66 | 525128564 | TCTGCAACTGATAGGGTCGC | CATGGTCGCTCGGTTTAACC | 98 | X | - | - | - | - | X | X | X |
| 2_1399 | 2H | 103.73 | 478645444 | CCGCCTGACCGAGAAATCT | AACGGCTTTCTCCTCCATGA | 62 | X | - | X | - | X | - | X | - |
| 2_0585 | 2H | 103.73 | 442060504 | GCTGAGATTGTTTGGTGCCA | TCATCTATGCTGCCACGACC | 110 | X | - | X | - | X | - | X | - |
| 2_1166 | 2H | 108.71 | 517863521 | CATCAACTACCGCTGCAAGT | ATCGATTGGCTCCTCACTGT | 97 | X | - | X | - | X | - | X | - |
| 2_1144 | 2H | 113.28 | 527707555 | CCCTCATCAAGCAGGACGAT | ACCCTTTCTTCTATTTACGCAGC | 97 | X | ND | ND | ND | X | ND | ND | ND |
| 2_0528 | 2H | 118.78 | 555019117 | ATGGTCACCCTGGAGATGTG | CCATCAGCAGAAGTCTTGAAGA | 77 | X | ND | ND | ND | - | ND | ND | ND |
| 1_1214 | 2H | 133.59 | 577523382 | CGCAGTGAAGGCAGGGTATA | CCACGTCCATTGTTCAGTCA | 77 | X | ND | ND | ND | - | ND | ND | ND |
| 1_0823 | 2H | 133.59 | 577651121 | AGGCCTCATCAGTAGGAAGC | AGGGACTCACATGATGCGAT | 87 | X | ND | ND | ND | - | ND | ND | ND |
| 2_0715 | 2H | 213.08 | 646502374 | TGCAGCCAGTGGACTTAATG | GATTTGCTCCACACAACGGT | 87 | X | ND | ND | ND | - | ND | ND | ND |
| 1_0551 | 2H | 221.70 | 651696832 | GTAGCTGATACCTGAGAGCCA | CTCATGCATCAAAGGCGTCAT | 68 | X | ND | ND | ND | - | ND | ND | ND |
| 1_1002 | 3H | 68.06 | 39043272 | TACCTGGCCAATTCCTCCAA | CTGTGATGATTCCTGCGACA | 103 | X | - | X | - | - | X | - | X |
| 2_1101 | 3H | 74.51 | 54523288 | GCTTGGCAGATTCCTCCTTG | CTCCGATGCAGCAAGATGAC | 129 | X | X | X | - | - | - | - | X |
| 1_0380 | 3H | 78.82 | 125195845 | ACAGAACCAAATATCAGCAGCT | AAATAATAACGTCGTGGTTGCG | 59 | X | X | - | X | X | X | - | X |
| 2_1129 | 3H | 79.88 | 181511027 | GGAAGAACTGCACCAAGACG | GAGAGCTTGAACTTGCGGAG | 124 | - | - | - | - | - | - | - | - |
| 1_1258 | 3H | 79.88 | 179020686 | TGCCTCATCAACTAGCAGTACT | CAGTACGATTGATCACACCAGC | 133 | X | X | - | X | X | X | - | X |
| 2_0288 | 3H | 82.03 | 337603488 | ACGAGAGCAGGAGATAAGGA | CCATCATCAAAGCGGGCC | 150 | X | X | - | - | X | X | - | - |
| 2_0002 | 3H | 82.03 | 376617629 | TACCCGCGCTATGACTCAAA | CCGGTGGTTCAATACTTGTCC | 121 | X | X | - | - | X | X | - | - |
| 1_0966 | 3H | 82.03 | 383547550 | CCTTTGCACTTGTTGAGAAGC | ATACACCAGAGGCAGCATCC | 162 | X | X | - | - | X | X | - | - |
| 1_0620 | 3H | 82.03 | 380612661 | ACAATAACAGAGAAGCCCACA | TCGTTTGGAACTGGTAGGACA | 77 | X | X | - | - | X | X | - | - |
| 2_1472 | 3H | 84.19 | 339775259 | CGGAGTGAAGGTGTTAATCGTC | GACTCCACCCTGCTCGTAAC | 124 | X | X | - | - | X | X | - | - |
| 2_1435 | 3H | 84.19 | 369794368 | GAAGCAGGGAGACGGTAAGA | GAAACAGACTCGAAATGGGGA | 94 | X | X | - | - | X | X | - | - |
| 2_0801 | 3H | 84.19 | 348705578 | TGTCGGTCTGAGTCTGATGT | ACAAACTGCAGCAACAAAGC | 81 | X | X | - | - | X | X | - | - |
| 2_0583 | 3H | 84.19 | 333981543 | CGTTTCCTCTTCAATCGCTGA | TGGGGATCTCATATACGTCACG | 103 | X | X | - | - | X | X | - | - |
| 2_0486 | 3H | 84.19 | 384118435 | AAAGACGGTTGAAGGAAGCC | GTCTGCAGCCATGTTAACACT | 116 | X | X | - | - | X | X | - | - |
| 1_1530 | 3H | 84.19 | 350149112 | KASP Assay |  |  | X | X | - | - | X | X | - | - |
| 1_1283 | 3H | 84.19 | 337029655 | KASP Assay |  |  | X | X | - | - | X | X | - | - |
| 1_1125 | 3H | 84.19 | 371062653 | GGGTAAGAAGAGTCCGTGCT | CAGCACCAACTCACGAAACA | 75 | X | X | - | - | X | X | - | - |
| 1_0925 | 3H | 84.19 | 379139774 | AGGTCATCAATAGTACAACCACA | GTGAGCAGCAGATGTAGCAC | 65 | X | X | - | - | X | X | - | - |
| 1_0926 | 3H | 85.26 | 398121010 | GGAAAGTAATGGAGAAGCTGGAG | GCCACAGACTCCTTACTCGT | 88 | X | - | - | - | X | - | - | - |
| 1_0224 | 3H | 85.26 | 390615716 | ATCCCACCGATTACAGGGC | CCATTGGTGCAGAGACGTAA | 100 | X | X | - | - | X | X | - | - |
| 1_0225 | 3H | 89.73 | 411660833 | TGTCCATGAGCCAGAGATACT | CAAATCTGGCCGTGCTTATCA | 84 | X | - | - |  | X | - | - | - |
| 1_0653 | 3H | 92.55 | 439953129 | GTCAGAGTCAGACGGGCTAA | ATGTCCACCCATCTTACCGC | 98 | X | - | X | - | X | - | X | - |
| 1_0281 | 3H | 98.41 | 455306801 | TGCGATGTTCAGTCCTCGT | ACAGAAGGTGAAGCAGGC | 114 | X | - | X | - | X | - | X | - |
| 1_1314 | 3H | 107.40 | 485299247 | CTGATTTGCCAAACAGTCGAAT | ACTTGTTGCATGCTGTCTGTTC | 113 | X | X | X | - | X | X | X | - |
| 1_1241 | 3H | 108.07 | 489339448 | CTCTCGCTGGGCACGATG | GACGCCTTCTACAAGTCGGT | 143 | X | - | X | - | X | - | X | - |
| 2_0597 | 3H | 119.10 | 502544377 | CAGGCAGGATGGGAAGTCG | ACTCACGGAACTCTTGACGT | 101 | X | - | - | - | X | - | - | - |
| 2_0362 | 3H | 119.10 | 501969690 | ACCAAGGTAAGATTCAGCAGTC | AACAACAGATGGCAAGCAGT | 88 | X | - | - | - | X | - | - | - |
| 2_1083 | 3H | 156.06 | 549663644 | CCCACAAGACAGAACCACAC | CCGACGCCATCCTATCTCAA | 89 | X | - | X | - | X | - | X | - |
| 2_1381 | 3H | 157.14 | 550857032 | TCTCCGACAGAAGGTAGTCA | TGCATCCACCATTAGCCTTTG | 106 | X | - | X | - | X | - | X | - |
| 2_1495 | 3H | 160.37 | 553489246 | CAATGCACAAGGGCTGAACA | ACTGCTGAGAAACAACCTAGTG | 87 | X | - | - | - | X | - | - | - |

^1^ Genetic position according to Druka et al. 2011. ^2^ Physical positions converted according to Morex V2 assembly (Monat et al. 2019). ^3^polymorphic for the corresponding marker in the population,^4^not determined, ^5^not polymorphic.

**Suppl. Table S2**. Markers used for identification of F_1_ plants.

| SNP | Chr. | Genetic position (cM)^1^ | Physical position (bp)^2^ | | Primer sequences 5’ to 3’ | | Size (bp) |
| --- | --- | --- | --- | --- | --- | --- | --- |
|  |  |  |  |  | Forward | Reverse |  |
| 1_0576 | 7H | 41.85 | 43935880 | CGAAACGACAATTCCGGAGA | | CGCCATAGATGCCAACAACA | 107 |
| 2_1302 | 7H | 79.6 | 341741457 | CCAAAGCTAGGAACGCAACA | | CCCGGCTCACTGTAGATGAT | 85 |
| 2_0103 | 7H | 102.85 | 573448717 | ATTCTGGCTTGCCGATGATC | | GTAGCCGCCGACATCCTC | 64 |
| 2_1145 | 3H | 42.47 | 36392076 | CTCATGGCCTTCGACAGC | | ATCACCATCGGCCTCTTGTG | 131 |
| 2_0798 | 1H | 55.49 | 356693611 | GGTGTCATTATTCATATGTGCAAGG | | GCAGAAAGCGTATGGAGACTC | 144 |

^1^ Genetic position according to consensus map (Druka et al. 2011).

^2^ Physical positions converted according to Morex V2 assembly (Monat et al. 2019).

**Suppl.** **Fig. S1** Cluster analysis of original *ert-d* mutants with respect to plant height (PH) and spike density. Plant height was measured from surface of the soil to the top of the spike excluding awns. Spike density was calculated as the number of kernels per length of spike.


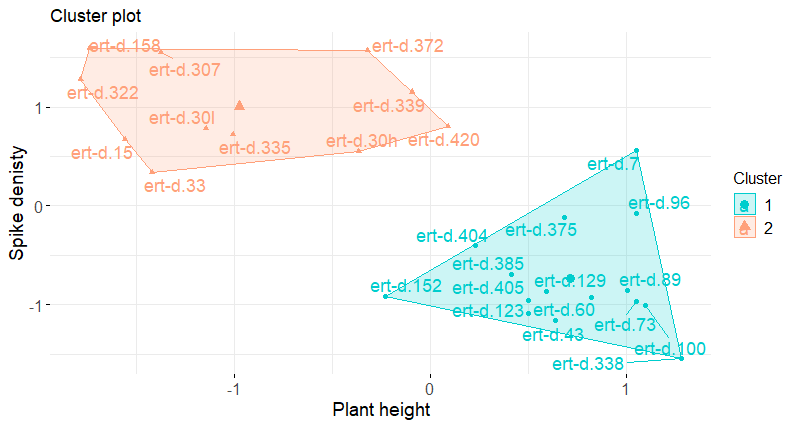

Supplement: Supplementary file 1 — Supplementary file1 (DOCX 96 kb) [file 425_2021_3653_MOESM1_ESM.docx]
